# Study on the adsorption properties of methyl orange by natural one-dimensional nano-mineral materials with different structures

**DOI:** 10.1038/s41598-021-90235-1

**Published:** 2021-05-20

**Authors:** Lijuan Wu, Xuewen Liu, Guocheng Lv, Runliang Zhu, Lintao Tian, Meng Liu, Yuxin Li, Wenxiu Rao, Tianming Liu, Libing Liao

**Affiliations:** 1 Beijing Key Laboratory of Materials Utilization of Nonmetallic Minerals and Solid Wastes, National Laboratory of Mineral Materials, School of Materials Science and Technology, China University of Geosciences, Beijing, 100083 China; 2 CAS Key Laboratory of Mineralogy and Metallogeny/Guangdong Provincial Key Laboratory of Mineral Physics and Material, Guangzhou Institute of Geochemistry, Chinese Academy of Sciences, Guangzhou, 510640 China

**Keywords:** Environmental sciences, Solid Earth sciences

## Abstract

Methyl orange (MO) is a common anionic azo dye that is harmful to the environment and biology, so it must be treated innocuously before it can be discharged. Adsorption is an effective method to remove anionic dyes. Nanotube mineral is a natural one-dimensional adsorption material, and its unique morphology and structure endow it with good adsorption capacity. Although there are many related studies, there is a lack of in-depth discussions on the influence of nanotube’s composition and structure on the adsorption of dyes and other pollutants. In this paper, two kinds of natural one-dimensional silicate minerals [halloysite nanotubes (HNTs) and chrysotile nanotubes (ChNTs)] with similar morphology but slightly different compositions and crystal structures were used as adsorbents, and MO solution was used as simulate pollutants. It is the first time to discuss in depth the influence of the composition and structure of nanotube minerals on their charge properties and the adsorption performance of methyl orange dyes. It is found that HNTs and ChNTs have different adsorption capacity due to the difference of electronegativity between Al^3+^ and Mg^2+^ in the crystal, so they possess negative and positive charges respectively in near-neutral solution, which leads to the adsorption capacity of MO by ChNTs with positive charges which is greater than that of HNTs.

## Introduction

**Figure 1:**
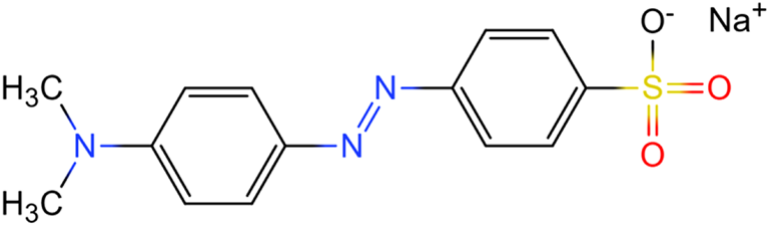
Schematic diagram of molecular structural formula of methyl orange.

The emergence of synthetic dyes has led to a chemical dye revolution^1^ and made the world more colorful. In the early twentieth century, synthetic dyes gradually have replaced natural dyes and played a dominant role in the dye field because of their advantages such as easy synthesis, good coloring, non-fading, wide application and so on^2^. With the expansion of production scale and application fields, synthetic dyes have gradually infiltrated into all aspects of life^3^. While they have brought colorful colors, synthetic dyes also bring serious water pollution problems. Dye wastewater produced by textile, paper, leather and other industries has become one of the main sources of water pollution^4–6^. The synthetic dye wastewater is mainly composed of organic components, which are complex and easy to show color in water, and usually slow or difficult to degrade in the natural environment^7,8^. Among the synthetic dyes, anionic azo dyes account for half of the dye synthesis and industrial application^9^. Due to the low coloring rate on natural fibers, anionic dyes account for a large proportion of the dye wastewater discharged by printing and dyeing factories. Methyl orange [(MO) dimethylaminoazobenzenesulfonate] is a common and typical azo anionic dye. This water-soluble organic synthetic dye has very high colorability and presents a bright orange color when dissolved in water. As shown in Fig. 1, azo dyes such as methyl orange contain aromatic and –N = N– groups in their molecules, which are highly toxic, carcinogenic and teratogenic^10,11^, and are harmful to the environment and organisms^12,13^. MO is selected as a simulated pollutant in this study, and its molecular diameter is estimated to be about 6–8 nm according to its molecular weight and structure.

In addition, the dyes in the wastewater can lead to the deterioration of water quality^14^, so the wastewater containing dyes must be treated innocuously and the dye components need to be removed in order to discharge to the natural water environment or carry out secondary use^4,15^. There are many treatment methods for dye wastewater, such as flocculation sedimentation method, membrane separation method, oxidative degradation method, etc^16,17^. There are many their own advantages, but a large amount of cost and complicated operation are often required, so it is difficult to popularize and apply on a large scale^18,19^. At present, adsorption technology is the most widely used method to remove dyes, which is favored by researchers because of its simplicity, safety, high efficiency, economy, wide applications and so on^15,20–23^. Adsorption is selective and can specifically remove target substances from complex pollutant solutions^24^. Some researchers even believe that adsorption may be one of the most effective ways to purify water sources^24–26^. Adsorbent is the core of adsorption technology, it has a wide range of sources and various types^22,23,27,28^. Mineral adsorbent is a popular research and application object in the field of adsorption. Because minerals are not only with low cost and large reserves but also with recyclable properties, so it is an ideal type of green adsorbent^29–31^. Natural nano tubular mineral is a kind of mineral adsorbent with good adsorption properties. Its unique morphology and structure make it to possess good environmental compatibility, large specific surface area, rich surface efficiency and high adsorption efficiency, so it is widely used in the removal of organic pollutants such as dyes^32–36^.

**Figure 2:**
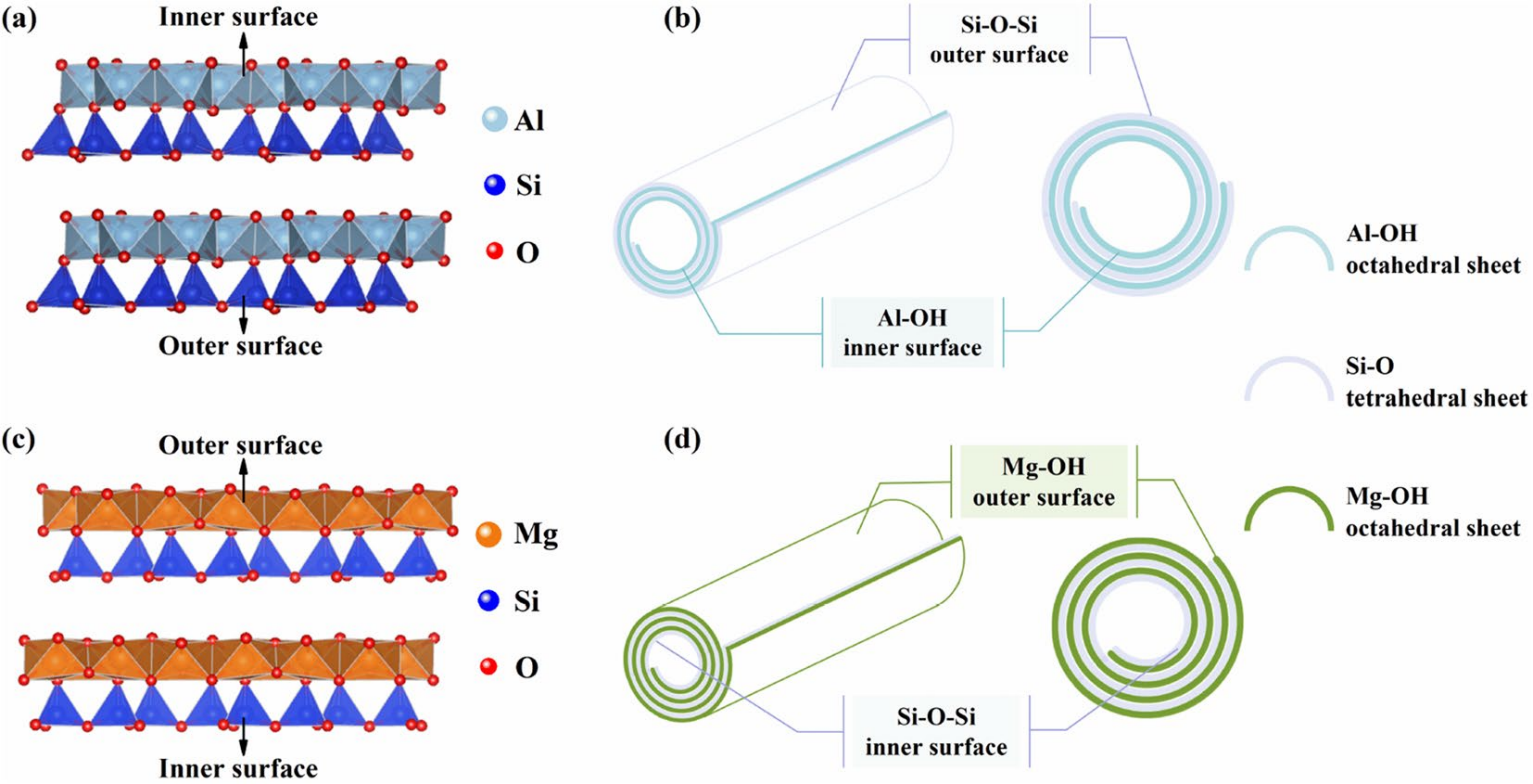
The crystal structure of kaolinite (**a**) and ChNT (**c**); the morphological diagrams of HNTs (**b**) and ChNTs (**d**).

Halloysite nanotubes and Chrysotile nanotubes are two kinds of one-dimensional nano tubular minerals with similar morphology but with slightly different composition and structure, and both of them belong to layered silicate minerals. Halloysite is a kind of layered silicate clay mineral composed of 1:1 Al_2_[Si_2_O_5_](OH)_4_ layers^37^, which belongs to the kaolinite group. Its chemical composition is similar to that of kaolinite, but compared with kaolinite, halloysite is usually hydrated, and each Al_2_[Si_2_O_5_](OH)_4_ cell can contain up to 2 H_2_O^38^. It is usually fibrous or tubular under electron microscope^39^. Due to the lack of HNTs single crystal samples needed for accurate calculation, the crystal structure data of HNTs can’t be found even in the latest ICSD^40^, so a reasonable crystal structure diagram of HNTs can’t be given. Therefore, the crystal structure of HNTs can only be indicated by the crystal structure of kaolinite with high similarity, as shown in In Fig. 2a the HNTs crystal structure, layered crystals are formed of Si–O tetrahedron and Al–OH octahedron^41^. Due to the existence of water molecules between layers, the strong hydrogen bonding system between the original layer and the layer is destroyed, and the difference between Si–O tetrahedron and Al-OH octahedron can be overcome by crimping^41^. This leads to the appearance of the crimped structural unit layer of HNTs with Si–O tetrahedron outside and Al-OH octahedron inside^41,42^ (as shown in Fig. 2a,b), forming nanotubes with an inner diameter of about 10–20 nm.

Chrysotile is also a 1:1 layered silicate mineral, which is composed of Mg_6_[Si_4_O_10_](OH)_8_ layers formed by the combination of Si–O tetrahedron and Mg-OH octahedron^43^, as shown in Fig. 2c. Because the translation period of O–O in the [SiO_4_] hexagonal grid of tetrahedron is different from that of O(OH)–O(OH) in octahedron, and there is incoordination between the two basic unit layers, a crimped shape of Mg-OH octahedron in the outer layer and Si–O tetrahedron in the inner layer is generated in ChNTs in order to overcome the disharmony between the two basic unit layers, and finally ChNTs present a fiber tube structure^41^, as shown in Fig. 2d. But the inner diameter of ChNTs is generally small, about 2–20 nm^41^. This curling condition is just the opposite of HNTs, which causes the outer surface of ChNTs to be positively charged in aqueous solutions^44^.

Natural nanotube adsorbents have been widely studied in the field of adsorption due to their unique physical and chemical properties. However, the current research on the adsorption of dyes by natural nanotube minerals mainly focuses on the modification of nanotubes and the mechanism of the adsorption process^35,45–47^, but there is a lack of in-depth discussion on the influence of composition and structure on the adsorption of dyes and other pollutants. Ionic pollutants in aqueous solutions always have some kind of charge, for example, methyl orange is an anionic dye, then the charged properties of mineral surfaces in water will naturally have a great impact on the adsorption effect of ionic dyes^48–50^. Therefore, the surface charge of minerals is one of the main reasons that affect the adsorption of anionic dyes by minerals. The composition and structure of the material determine the property. Therefore, the composition and structure of nanotube mineral structure are closely related to its charge properties in solution and the adsorption effect of MO dyes.

At present, the relationship among the composition and structure of the nanotube minerals, their charge properties, and adsorption performance has not been studied in depth. In this study, nanotube minerals that have similar morphologies but different compositions and structures were used for comparative adsorption of MO dyes. The difference in the removal effects of two natural one-dimensional mineral materials on MO dyes was explored through mineral characterization test and adsorption isotherm model fitting. It is the first time to discuss in depth the influence of the composition and structure of nanotube minerals on their charge properties and the adsorption performance of methyl orange dyes.

## Materials and methods

### Materials

HNTs were from China Kaolin Co., Ltd., Suzhou, China. The origin of ChNTs were Linqu County, Weifang City, Shandong Province, China. MO (C_14_H_14_O_3_N_3_SNa, AR) was purchased from Xilong Scientific Co., Ltd.

### Treatment of adsorbent

The purity of HNTs was high and no further treatment is needed. ChNTs were raw ore when purchased and needed to be purified and dispersed before they can be used. With the use of metal sharp-beaked tweezers, ChNTs were stripped from the original ore into a dispersed silk floc. Then they were soaked in beakers filled with distilled water. The beaker was put in the ultrasonic machine, for 1 h as to wash and disperse the samples. In the process of ultrasonic cleaning, the water needed to be changed several times to obtain the clean and dispersed silk floc ChNTs.

### Preparation of solution

The preparation of mother liquor: first of all, MO mother liquor with the concentration of 1000 mg/L is prepared for the adsorption experiment. 1000 mg MO was weighed, then it was added to the 1000 mL volumetric flask and fixed to the scale, and finally 1000 mg/L MO solution was obtained. Then the MO mother liquor was diluted to different concentrations. When the adsorbent was HNTs, the mother liquor is diluted to obtain MO solution with concentrations of 10, 30, 50, 80, 120, 150, 300 and 400 mg/L, respectively. When the adsorbent was ChNTs, the mother liquor was further diluted to solutions with different concentration gradients such as 10, 30, 50, 100, 150, 200, 300, 400 and 600 mg/L.

Preparation of standard solution: in order to obtain the linear relationship in accordance with Lambert–Beer law, different volumes of MO mother liquor were added into the colorimetric tube with pipette gun, the volume was fixed to the 25 mL scale with deionized water, and the standard solution diluted to 2, 4, 5, 8, 10 mg/L was obtained.

### Dye adsorption study

The adsorption effect of anionic dyes on adsorbents with similar morphology but different microstructure was studied. 20 mL MO solutions with different concentrations were placed in the centrifuge tube, and 0.05 g adsorbent was added respectively. The centrifugal tubes were sealed and oscillated in a constant temperature oscillator for 3 h at room temperature. After the oscillation was completed, the solution in the centrifuge tube was centrifuged to separate the adsorbent from the dye solution (10,000 rmp, 2 min). The supernatant after centrifugation was stored in the 20 mL sample bottle and kept away from light. After the solution was poured out, the adsorbent in the centrifugal tube was dried in a constant temperature blast drying box at 60 °C for 12 h, and the dried adsorbent was collected and preserved. After the supernatant was diluted in a certain proportion, the absorbance of the supernatant was measured at the characteristic absorption wavelength of 465 nm of MO solution by ultraviolet spectrophotometer. After comparing with the absorbance value of the standard solution, the concentration was obtained.

### Analysis methods

The analysis of crystal structures of HNTs and ChNTs was completed by X-ray diffractometer (XRD, Bruker Scientific Instruments Hong Kong CO., Ltd.), and the working conditions are as follows: using 40 kV and 100 mA Cu-Kα target as a radiation source, scanning speed 5°/min, scanning range 3° ≤ 2θ ≤ 70°. The micro-morphologies of HNTs and ChNTs were obtained by scanning electron microscope (SEM, JEOL Ltd.). The relevant data of specific surface area, pore size distribution and other adsorption properties of two kinds of natural minerals were obtained by BET specific surface area test method using automatic specific surface area and pore size distribution analyzer (BET, Quantachrome Instruments Co., Ltd.) and nitrogen as adsorbent. The Zeta potentials of HNTs and ChNTs were measured by Zeta potential analyzer (Malvern Instruments Co., Ltd.). The zero electric points of two kinds of minerals were determined by pH meter (Shanghai INESA Scientific Instrument Co., Ltd.). The change of the structure and composition of the adsorbent under different pollutant concentrations was characterized by Fourier transform infrared spectroscopy (FTIR).

## Results and discussion

### Morphology and characterization

**Figure 3:**
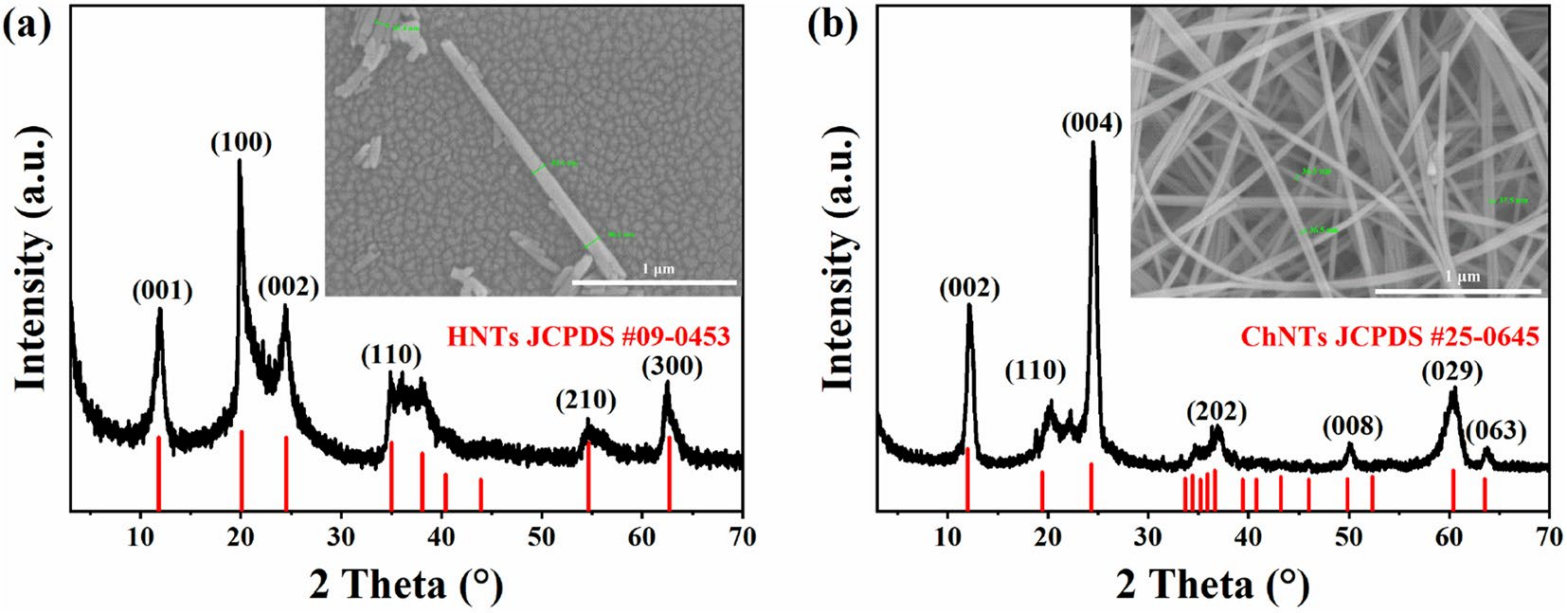
XRD pattern of natural mineral adsorbents: (**a**) HNTs; (**b**) ChNTs. Inset: SEM images of natural mineral adsorbents.

The structures of two kinds of natural mineral adsorbents were characterized by XRD, and the XRD patterns were shown by Fig. 3. According to Fig. 3a, there is a characteristic peak corresponding to the standard card JCPDS #09-0453 on the spectral line of HNTs, such as (001), (100), (002), (110), (210), and (300), etc., and the characteristic peak is sharp, which proves that the HNTs selected in the experiment has higher purity and better crystallinity. According to Fig. 3b, the characteristic peak of XRD line of ChNTs corresponds well to the standard card JCPDS #25–0645 at positions (002), (110), (004), (202), (008), (029), and (063), respectively. And the characteristic peak is significant and sharp, which indicates that the purified ChNTs have good crystallinity and high purity. From the SEM images of HNTs and ChNTs embedded in Fig. 3a and b, it can be seen that the diameter of HNTs is 50–100 nm, and the diameter of ChNTs is 30–40 nm. The length of HNTs is mainly about 1 μm, but not more than 2 μm. The length of ChNTs could not be estimated because of its bending and winding under electron microscope. The micro-morphologies of the two minerals are similar, and both of them are one-dimensional nanowire-like materials.

**Figure 4:**
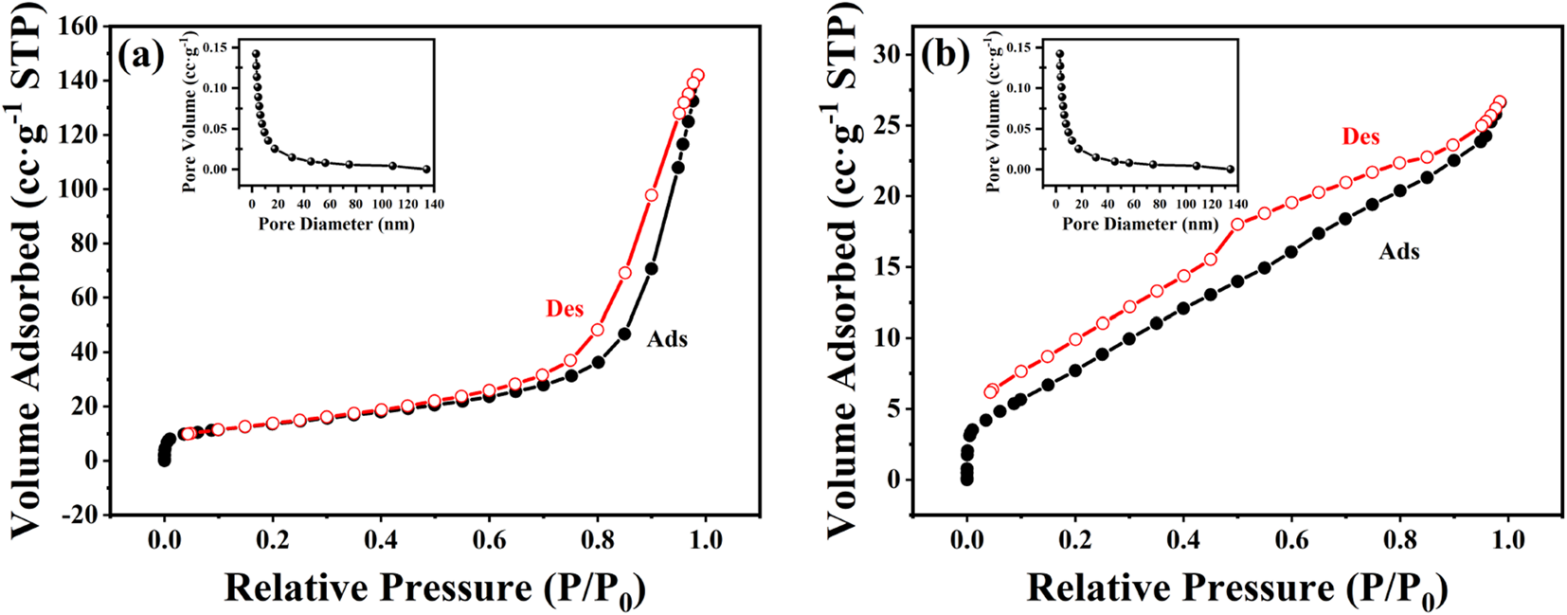
Adsorption–desorption curve of (**a**) HNTs and (**b**) ChNTs. Inset: Pore size distribution curve of (**a**) HNTs and (**b**) ChNTs.

The surface adsorption properties of HNTs and ChNTs were measured by BET specific surface area test. The results of BET test showed that the specific surface area, pore volume and average pore diameter of HNTs were 48.713 m^2^/g, 0.217 cc/g and 17.422 nm, separately; and those of ChNTs were 29.371 m^2^/g, 0.031 cc/g and 3.418 nm, separately. According to the BET theory, the N_2_ adsorption–desorption curve of HNTs is a type III isotherm (Fig. 4a), which indicates that the solid adsorbent may have a hydrophobic surface with multi-molecular layer adsorption, or the adsorption of adsorbents to adsorbates is weaker than the interaction between adsorbates themselves. Therefore, the adsorption capacity is less in the low-pressure region, indicating that the attraction between the adsorbent and the adsorbate is weak. When the relative pressure approaches 1, the adsorption capacity increases rapidly, indicating that there are fillable pores on the adsorbent, which is consistent with the tubular structure of HNTs. The N_2_ adsorption–desorption curve of ChNTs is a type II isotherm (S-type isotherm) (Fig. 4b), which may occur on the surface of non-porous solids or in the process of reversible adsorption of single multi-layer on macroporous solids. The curve is also consistent with the case of ChNTs.

**Figure 5:**
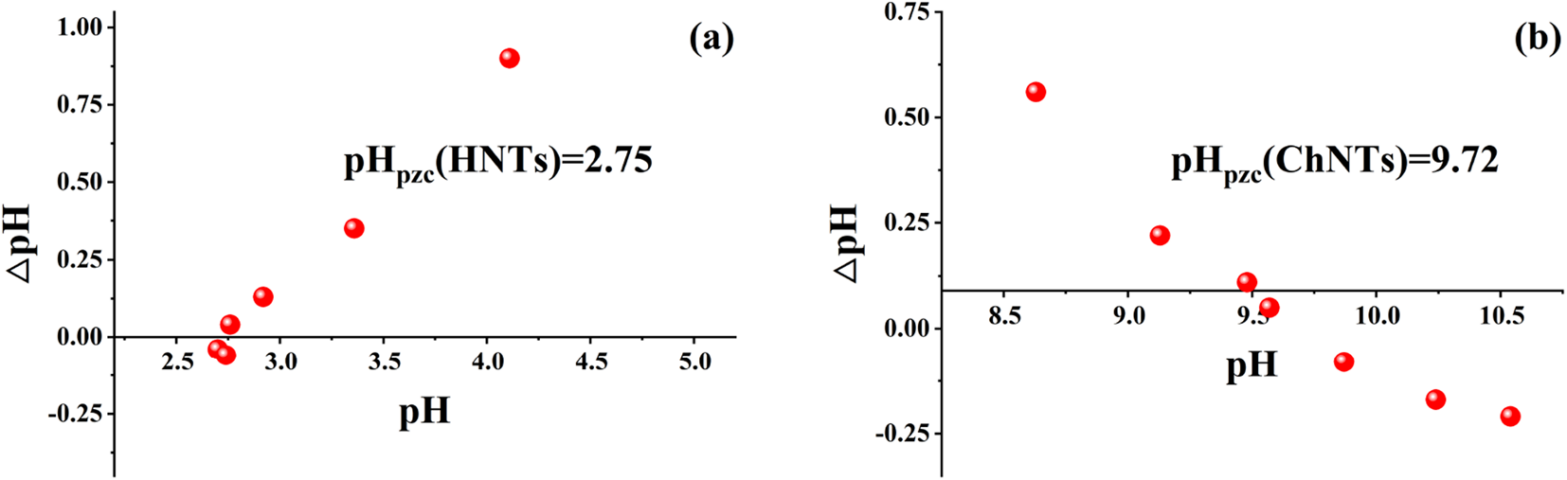
Point of zero charge of HNTs (**a**) and ChNTs (**b**).

The test results of Zeta potential analyzer showed that the Zeta potentials of ChNTs and HNTs were 3.45 mV and − 18.2 mV, respectively. It has been shown that the particles of HNTs have a negative charge and the particles of ChNTs have a positive charge in neutral solution. It has also been proved that the zero point test results of two natural minerals (Fig. 5). It is found that the pH_pzc_ of HNTs is 2.75 and the pH_pzc_ of ChNTs is 9.72. Based on the theory of colloidal chemical thermodynamical potential, the mineral surface has a negative charge when pH_pzc_ < pH and a positive charge when pH_pzc_ > pH. Therefore, in the solution with near neutral pH value, HNTs will show negative surface charge, which is more favorable for them to adsorb cations; while ChNTs will show positive surface charge, so they are easier to adsorb anions. There are three ways of charging clay: (1) the isomorphous replacement of ions in the lattice, (2) the valence bond fracture of the edges of particles, and (3) the ionization of humic acid on the surface of particles^51,52^. Pathways (1) and (2) are the main causes of clay with charge. Pathway (1) brings the permanent charge, and pathway (2) bring the variable charge. According to the XRD results, the clay minerals used in the experiment are all samples with high purity, and there is no drum-like characteristic peak of organic matter, so their charge does not come from the pathway (3). The isomorphic substitution of ions in the layered clay lattice results in a negative charge on the surface perpendicular to the c-axis, and the amount of this charge depends on the number of ion substitutions in the lattice. The phenomenon of isomorphic substitution in HNTs and ChNTs is less^53–56^, so pathway (1) is not the main reason for their electrification.

In the process of clay crystal dispersion, the edge or surface damage will produce a negative charge due to the bond breakage, so that the adsorption of H^+^ in different pH media makes the edge (parallel to the c-axis) with positive or negative charges. According to the variation of zeta potential with pH, HNTs and ChNTs mainly carry variable charges. From the c-axis projections of HNTs and ChNTs (see Fig. S1), there may be a large number of broken bonds at the edges of both. Both silicate minerals are crimped into a tube shape by the crystal structure unit layer, so the broken bonds are mainly distributed at the mouth of the nanotube and the edge of the inner and outer surface as shown in Fig. 2b,d. The difference of charged properties between HNTs and ChNTs in solution is closely related to their composition and structure. The composition of HNTs and ChNTs is very similar, and each cell contains the same amount of Si^4+^, O^2−^, OH^−^, and the only difference is that there are 4 Al^3+^ in HNTs and 6 Mg^2+^ in ChNTs. The difference in the electronegativity of the central atom of the octahedron leads to the difference in the mode of bond connection and ability to adsorb H^+^. As you can see from Figure S1a and Figure S1b, in HNTs, 1 Al^3+^ connects 2 O^2−^ and 4 OH^−^, simultaneously, each OH^−^ is connected to 2 Al^3+^, the O^2−^ on each Al is connected to 2 Al^3+^ and 1 Si^4+^, and each Si^4+^ is connected to 4 O^2−^. As you can see from Fig. S1c,d, in ChNTs, 1 Mg^2+^ connects 2 O^2−^ and 4 OH^−^, at the same time, but each OH^−^ is connected to 3 Mg^2+^, and O^2−^ on each Mg^2+^ is connected to 3 Mg^2+^ and 1 Si^4+^, and each Si^4+^ is connected to 4 O^2−^. It can also be seen from Fig. S1 that although the number ratio of the octahedral central element to Si is different in the two kinds of crystals, Al: Si = Mg: Si at the edge is 1:1. Therefore, when the bond is broken at the edge, there may be three negative charge sites on an Al–O–Si, including two Al-OH (Al–O) with − 1/2 valence and one Si–O bond with − 1 valence; there may be four negative charge sites on an Mg–O–Si, three Mg-OH (Mg–O) with − 1/3 valence and one Si–O bond with − 1 valence. The charged property of the whole clay-water system is determined by the algebraic sum (net charge) of the positive and negative charge carried by the clay. At the same pH value, the minerals with more negative charge sites are easier to adsorb protons (H^+^) in the solution, and one Mg-OH (Mg–O) has less charge than Al-OH (Al–O), so the net charge of ChNTs after adsorption of H^+^ is more likely to be positive. Therefore, in the weakly acidic MO solution with a pH close to 7, HNTs have the negative charges and ChNTs have the positive charge, and these charges are mainly distributed in the nanotube’s tube mouth, the surface edge, and the damaged part on the surface.

**Figure 6:**
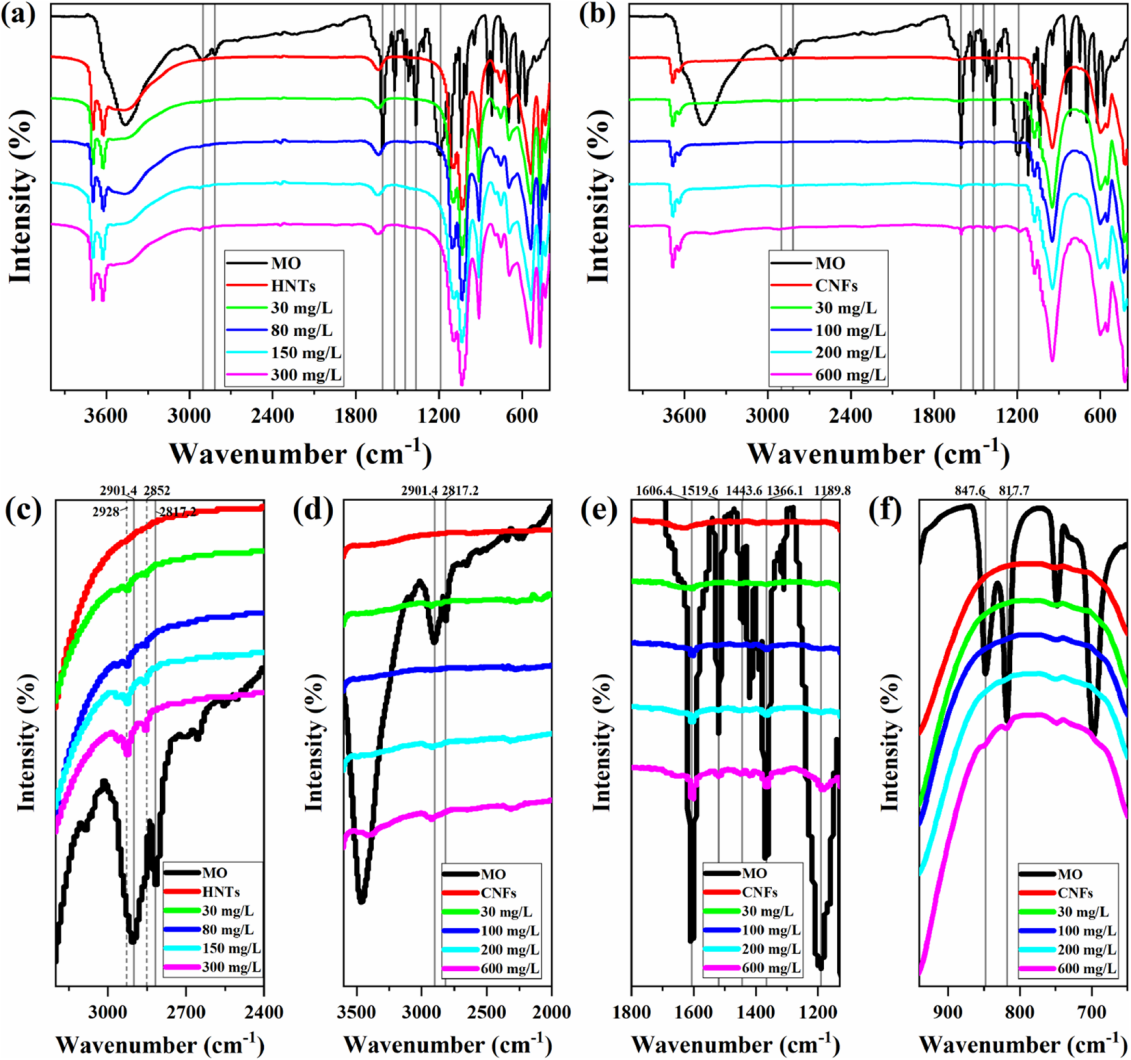
FTIR of HNTs (**a,c**) and ChNTs (**b,d,e,f**) at different initial concentrations.

The full and part infrared spectrum of the adsorbent before and after the adsorption reaction and MO dyes are shown in Fig. 6. The infrared data shows the characteristic absorption peaks of the functional groups on the MO molecule used in the experiment: the broad and strong peak at 1189.8 cm^−1^ is the absorption peak of the sulfonate functional group; 1606.4, 1519.6, and 1443.6 cm^−1^ are the vibrational absorption peaks of the benzene ring skeleton, 847.6 and 817.7 cm^−1^ are the out-of-plane deformation vibration absorption peaks of C–H of the two benzene rings in the MO molecule; the characteristic absorption peaks of –CH_3_ appear at 2901.4, 2817.2, and 1366.1 cm^−1^ (its absorption peak wave number is less than the ordinary –CH_3_ absorption peak wave number, –CH_3_ and N in the MO molecule are connected so the –CH_3_ absorption peak of MO shifts to the low wavenumber direction)^57,58^.

The intensity of the infrared absorption peak mainly depends on the type of transition, the polar intensity of the group, and the concentration of the analyte^58^. The stronger the polarity of the group, the greater the vibration absorption intensity, and the higher the concentration of the analyte, the greater the absorption intensity^58^. The adsorbents that adsorbed different initial concentrations of MO were compared with MO. It is found by comparison that with the increase of the initial concentration of the MO solution, the HNTs series samples were only changed that at nearly 2901.4 and 2817.2 cm^−1^ where the infrared absorption intensity of –CH_3_ has increased from scratch. From the enlarged detail of the HNTs series samples (Fig. 6c), it can be seen that the peak intensity of –CH_3_ has increased but the changes are small, and these indicate that HNTs adsorbed a smaller amount of MO. And compared with MO, after MO adsorbed by HNTs, wavenumber of the –CH_3_ absorption peaks in the infrared spectrum of the sample became larger, which may be caused by the interaction between HNTs and MO.

In the infrared spectrum of ChNTs adsorbing MO (Fig. 6b), the infrared absorption peaks in multiple wavenumber ranges have changed, and these details are shown in Fig. 6d–f. The detailed drawings show that with the increase of the initial concentration of MO, the infrared absorption peak intensity of various MO functional groups on the spectra of ChNTs series samples has increased to varying degrees, which indicates that ChNTs can adsorb MO. Comparing the infrared spectra of HNTs and ChNTs, it is not difficult to see that the adsorption effect of ChNTs on MO is better.

### Adsorption effect of natural one-dimensional nano-mineral materials on anionic dyes

**Figure 7:**
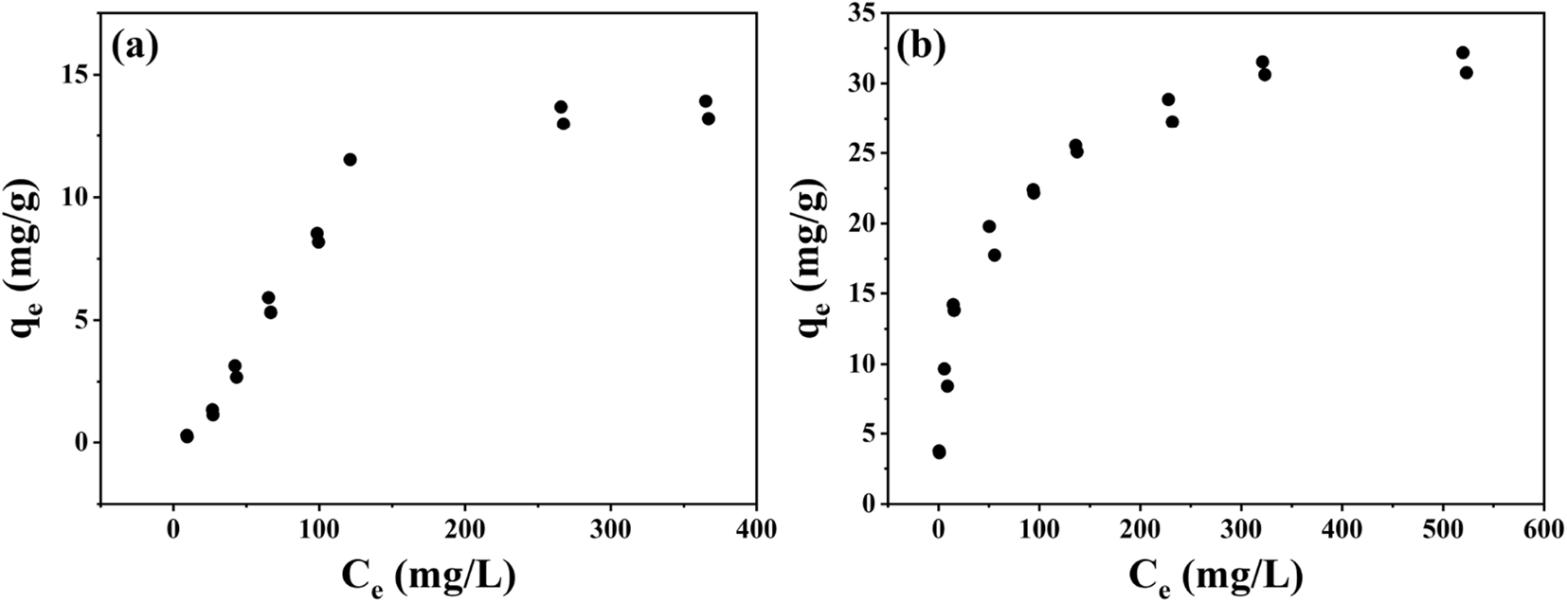
Adsorption isotherms of HNTs (**a**) and ChNTs (**b**). (Ce is the concentration of MO solution after the adsorption reaction reaches equilibrium, q_e_ is the adsorption amount of MO when the adsorption reaction is equilibrium.)

In order to directly compare the adsorption difference of anionic dye (MO) between HNTs and ChNTs, the adsorption experiments of MO dyes were carried out with two kinds of one-dimensional mineral materials, and the experimental data were fitted and analyzed by adsorption isotherm equation. The results show that the average maximum adsorption capacity of HNTs and ChNTs to MO dyes is 13.56 mg/g and 31.46 mg/g, respectively (Fig. 7). There is a significant difference in the amount of MO dyes adsorbed by the two adsorbents. This difference in adsorption capacity seems to be consistent with our original assumption. Although the micro-morphology of HNTs is similar to that of ChNTs, there is a significant difference in the adsorption effect of the same anionic dyes.

**Figure 8:**
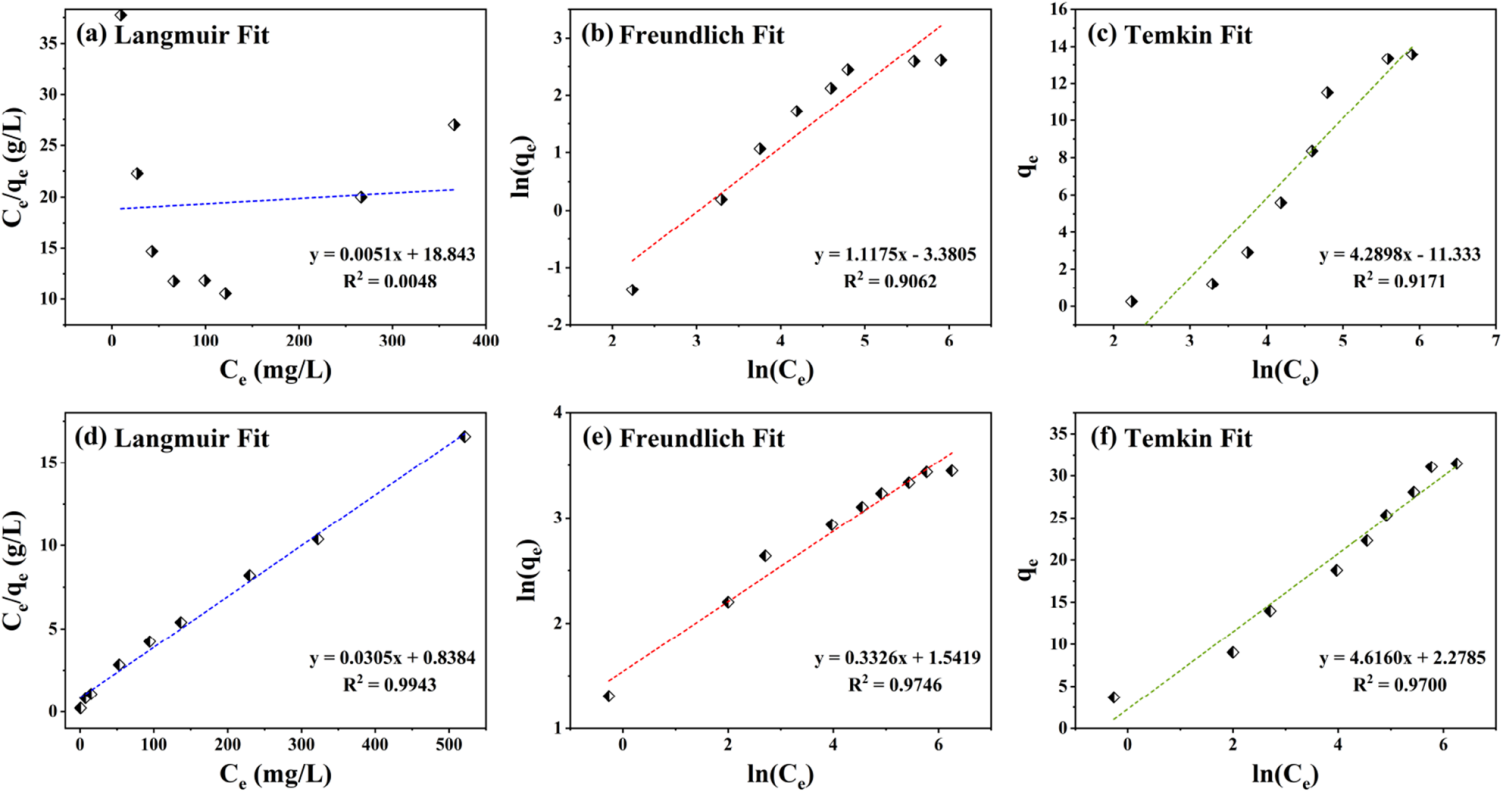
Linear fitting graphs of Langmuir (**a**), Freundlich (**b**) and Temkin (**c**) equations for HNTs adsorption of anionic dyes. And Linear fitting graphs of Langmuir (**d**), Freundlich (**e**) and Temkin (**f**) equations for ChNTs adsorption of anionic dyes.

**Figure 9:**
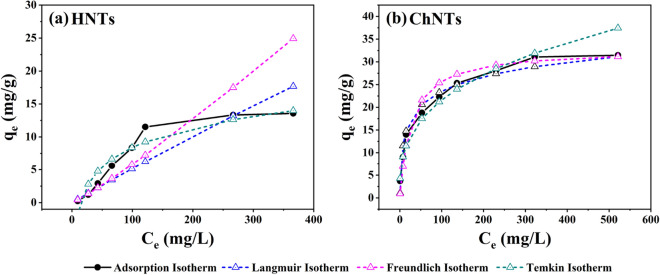
The adsorption model equation fitting isotherm of HNTs (**a**) and ChNTs (**b**) adsorption of MO dye.

In order to further reveal the reasons for the differences, we fit the data. In this work, three adsorption models are applied, which are Langmuir, Freundlich and Temkin respectively. Their equations are listed in Table 1^59,60^. According to the equation of adsorption model, the experimental data were fitted and the parameters are shown in Table 2. The isotherms of HNTs and ChNTs on MO dyes are shown in Fig. 7, and the model fitting isotherms are shown in Figs. 8 and 9. The Langmuir model represents a monolayer adsorption model, while both Freundlich and Temkin models are multi-layer adsorption theoretical models.

**Table 1:** Adsorption isotherm models for MO dye removal using HNTs and ChNTs^59,60^.

Model	Equation
Langmuir	$\frac{{C_{{\text{e}}} }}{{q_{e} }} = \frac{1}{{q_{m} K_{L} }} + \frac{{C_{e} }}{{q_{m} }}$ (1)
	$$R_{L} = \frac{1}{{1 + K_{L} C_{i} }}$$ (2)
Freundlich	$\ln \, q_{e} = \ln \, q_{F} + \frac{1}{n}\ln \, C_{e}$ (3)
Temkin	$q_{e} = A\ln K_{t} + A\ln C_{e}$ (4)

**Table 2:** Isotherm parameters of HNTs and ChNTs removing MO.

	Langmuir				Freundlich			Temkin		
	q_m_ (mg/g)	K_L_ (L/mg)	R^2^	Range R_L_	q_F_ (mg^(1–1/n)^L^1/n^g^−1^)	1/n	R^2^	K_t_ (L/g)	A	R^2^
HNTs	13.56	0.0003	0.0048	0.9023–0.9973	0.0340	1.1175	0.9062	0.0712	4.2898	0.9171
ChNTs	31.46	0.0364	0.9943	0.0438–0.7333	4.6735	0.3326	0.9746	1.6382	4.6160	0.9700

In the Langmuir model, when the separation factor R_L_ approaches 1, the adsorption effect is not ideal. In the experiment of adsorption of MO dye by HNTs, the value of R_L_ is between 0.9023 and 0.9973, so the Langmuir model may not be able to describe the adsorption process well. In addition, the R^2^ of Langmuir model (R^2^ = 0.0048) is much lower than that of Freundlich (R^2^ = 0.9062) and Temkin (R^2^ = 0.9171) model, so Langmuir model can’t describe the adsorption process. For this reason, the adsorption of MO dyes by HNTs does not belong to monolayer adsorption. The Temkin equation is an empirical equation, which is based on the homogeneity of adsorption heat on the surface of the adsorbent^61–63^. The Temkin equation assumes the adsorption heat decreases linearly when the adsorption coverage increases^64–66^, and the binding energy is evenly distributed around the place before reaching the saturated adsorption (the maximum binding energy), which is suitable for the multi-molecular layer adsorption process on the inhomogeneous surface^62,67^. Among the three models, the R^2^ of Temkin model is the largest, so the adsorption result is the most similar to Temkin model. The isotherms fitted by the models in Fig. 9a also proved that Temkin isotherm was in good agreement with the adsorption curves. This shows that the adsorption of HNTs for MO dyes is a multi-layer adsorption process, and HNTs may be a kind of adsorbent with heterogeneous surface and its surface adsorption heat distribution is uniform.

Current research has proved that HNTs have capillarity in solution^68,69^, so we think that the capillary effect plays a role in the adsorption of MO dyes by HNTs. The so-called capillarity is actually an interface phenomenon realized by additional pressure. The BET results show that the average pore diameter of the HNTs used in the study is only 17.422 nm. The diameter of MO molecules is about 6–8 nm smaller than that of HNTs, MO can enter the inside of HNTs. The inner surface of HNTs is composed of Al–OH. Since it is impossible to directly measure the wettability of the aqueous solution on the inner surface of HNTs, boehmite [γ-AlO(OH)] with Al–OH was selected as the outer surface as a substitute. The contact angles θ of the minimum and maximum concentrations (10 mg/L and 400 mg/L) of MO solution on the surface of Al–OH were measured, which were 15° and 19.5°, respectively (the contact angles are shown in Fig. S2). The two θ < 90° indicate that the MO solution in the experimental concentration range is moist to the inner surface of the nanotube. According to the Eq. (4) (in SI), when the contact angle θ < 90° (that is, the liquid wets the capillary), cos θ > 0, then ∆p > 0. ∆p > 0 means that the liquid will enter the capillary under additional pressure and move along the capillary to the inside of the capillary. Because the tube mouth of HNTs is negatively charged in MO solution, and MO is an anion, there is electrostatic repulsion between them. To compare the additional pressure and electrostatic repulsive force, the surface tension α of MO solution was measured by the capillary method^70^ (the experiment is shown in Fig. S3), and the additional pressure of 10 mg/L and 400 mg/L MO solution was calculated according to the Laplace formula. And the electrostatic repulsive force between nanotube and MO molecules was estimated. All detailed calculation processes can be found in supporting information. It is calculated that the additional pressure of 10 and 400 mg/L MO solution in HNTs is 6.8227 × 10^6^ N and 6.3585 × 10^6^ N respectively, and the electrostatic force of a single HNT to 10 and 400 mg/L MO is 1.6139 × 10^–8^ N and 5.6754 × 10^–7^ N. The additional pressure is much greater than the repulsion between the same charges. Under the action of additional pressure, MO dyes overcome electrostatic repulsion and enter into HNTs. However, because the internal space of HNTs channel is limited and the molecular size of MO is large, HNTs can only absorb a small amount of MO.

In the experiment of ChNTs adsorption of MO dyes, the R^2^ values of Langmuir, Freundlich and Temkin models are 0.9943, 0.9746 and 0.9700 respectively, and the three models have a high degree of fitting for the adsorption process. However, from either the value of the coefficient of determination R^2^ or the coincidence degree between the fitting line of each model and the adsorption isotherm (Fig. 9b), it is clear that the adsorption experiment is more consistent with the Langmuir equation. And the R_L_ (R_L_ = 0.0438–0.7333) between 0 and 1 also shows that the Langmuir model is favorable for the adsorption reaction, and the Langmuir model is a monolayer adsorption model. The average pore diameter of ChNTs is only 3.418 nm, less than the molecular diameter of MO, so it is difficult for MO to enter the narrow channel of ChNTs, so we think that the capillary force of ChNTs does not play a practical role during adsorption. It is known from the previous article that the charge on the surface edge and tube mouth of ChNTs is positive, so the process of monolayer adsorption of MO by CHNTs depends on electrostatic attraction.

## Conclusions

HNTs and ChNTs that are natural one-dimensional nano-mineral materials were used as adsorbents for the removal of MO dyes from water. In the near-neutral aqueous solution, the average maximum adsorption capacity of HNTs and ChNTs to MO dyes are 13.56 mg/g and 31.46 mg/g, respectively, and the latter is about twice as much as the former. Further adsorption isotherm model fitting study shows that the process of HNTs adsorption of MO dyes is more consistent with the Temkin model, which belongs to multi-molecular layer adsorption. The analysis results of BET adsorption–desorption curve also show that multi-molecular layer adsorption is easy to take place on the surface of HNTs. The adsorption of MO dyes by ChNTs is most consistent with the Langmuir model, so monolayer adsorption is the main adsorption process.

Although ChNTs also have a fibrous tubular structure, because of their small inner diameter, it is difficult for macromolecular dyes to enter the channel with the effect of capillaries, so ChNTs mainly absorb MO dyes by electrostatic gravity. In the condition that the specific surface area, average pore size and pore capacity of ChNTs are far lower than that of HNTs, the adsorption amount of MO dyes by ChNTs is still much higher than that of HNTs, which shows that the positive surface charge of ChNTs plays a major role in the adsorption process. The infrared test results also confirmed indirectly that there was a difference in the adsorption capacity. The above results show that HNTs and ChNTs, the one-dimensional nanotube materials with similar morphology, can obtain different surface charges due to the different curling forms that form the crystal structure, which leads to significant differences in their adsorption effects on MO anionic dyes.

## Supplementary Information

Supplementary Information.
