# Supplementary material for: Study on the adsorption properties of methyl orange by natural one-dimensional nano-mineral materials with different structures
Source: Sci Rep. 2021 May 20;11:10640. doi: 10.1038/s41598-021-90235-1 (PMC8138017; doi:10.1038/s41598-021-90235-1)
Supplement: Supplementary file 1 — Supplementary Information. [file 41598_2021_90235_MOESM1_ESM.pdf]

Scientific Reports

Study on the adsorption properties of methyl orange by natural one-dimensional nano-mineral materials with different structures

Lijuan Wu[1], Xuewen Liu[1], Guocheng Lv[1,*], Runliang Zhu[2], Lintao Tian[1], Meng Liu[1], Yuxin Li[1], Wenxiu Rao[1], Tianming Liu[1], Libing Liao[1,*]

[1] Beijing Key Laboratory of Materials Utilization of Nonmetallic Minerals and Solid Wastes, National Laboratory of Mineral Materials, School of Materials Science and Technology, China University of Geosciences, Beijing 100083, China.
[2] CAS Key Laboratory of Mineralogy and Metallogeny/Guangdong Provincial Key Laboratory of Mineral Physics and Material, Guangzhou Institute of Geochemistry, Chinese Academy of Sciences, Guangzhou 510640, China.
*email: guochenglv@cugb.edu.cn; lbliao@cugb.edu.cn.

Supporting Information

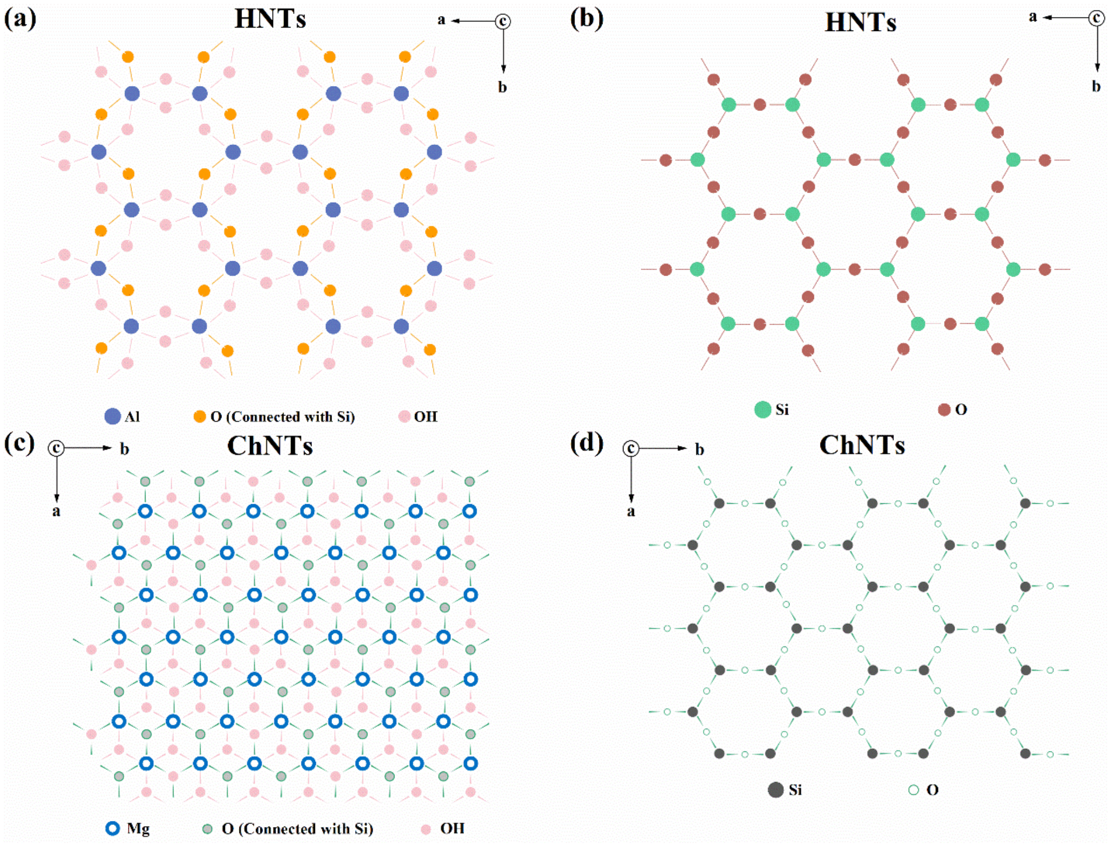

Figure S1. C-axis projection of the structural unit layer of HNTs and ChNTs crystals.

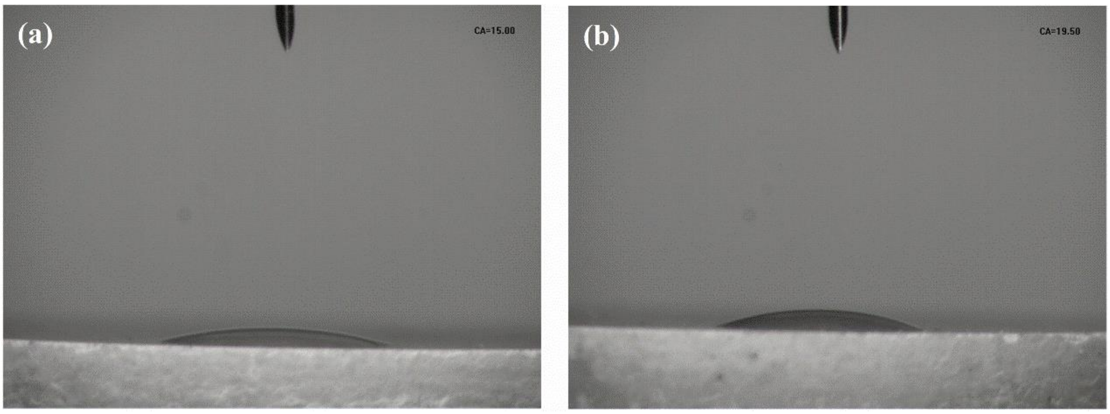

Figure S2. The contact angle θ of different concentrations of MO: (a) 10 mg/L and (b) 400 mg/L.

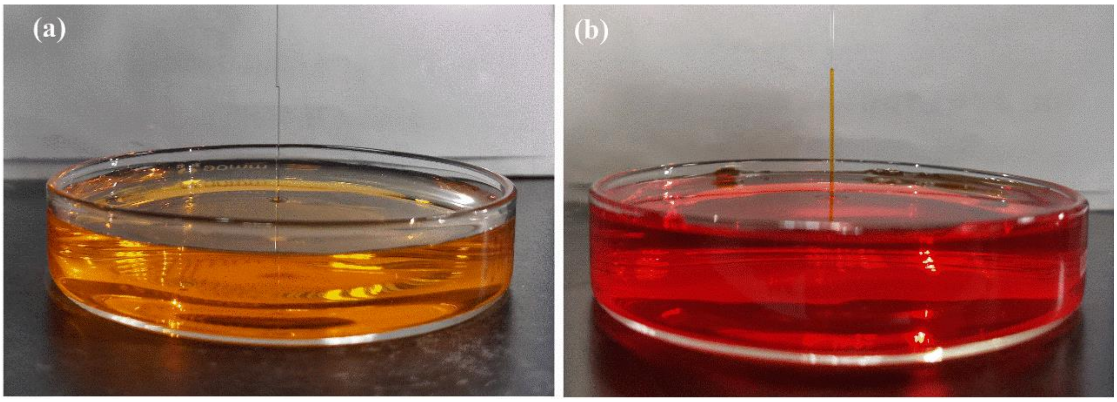

Figure S3. Measurement of surface tension at 10 mg/L and 400 mg/L MO by capillary method.

Supporting Information S1: Calculation of additional pressure and electrostatic repulsion

In order to facilitate discussion and calculation, we put forward the following assumptions:

① Each HNT is assumed to be a perfect hollow cylinder, and its lumen is also a cylinder.

② According to the results of SEM and BET, to facilitate the calculation, it is assumed that the length of an HNT is 1200 nm, the outer diameter is 80 nm, and the inner diameter is 20 nm.

③ In the calculation of electrostatic force, it is assumed that the solution that can be entered into the halloysite nanotube is cylindrical with the same shape and volume as the lumen of HNT. Secondly, in the calculation, HNT and this part of the solution are regarded as independent point charges respectively. Finally, a model is built. It is assumed that the liquid is located in front of the HNT's mouth and is in contact with the tube mouth, so the distance between the two charges which is needed to calculate in the electrostatic force that can be set to half the length of the cylindrical liquid (that is, half the length of the HNT), as shown in Figure S4.

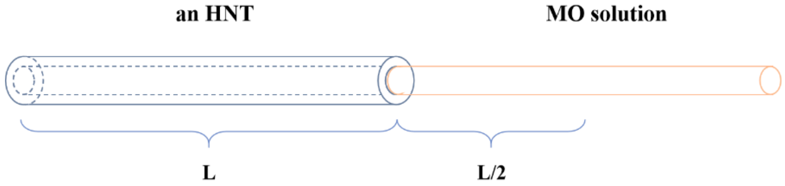

Figure S4. Model diagram of MO solution and an HNT.

④ The cation exchange capacity (CEC) of HNTs is 2-60 mmol/100g, the CEC of HNTs is assumed 40 mmol/100g.

1. Calculation of electrostatic repulsion

(1) The estimate of the weight of an HNT

According to assumptions ②

$R_{outside}$ = 40 nm, R_{inner}= 20 nm

According to the results of XRD, the layer spacing d_c of the c-axis of HNTs used in the experiment is 7Å.

If the number of crimp layers of HNT is b, then:

$b = (R_{outside} - R_{inner}) \div d = (40-20)\ nm \div 0.7\ nm \approx 43$

Because the HNT curls around the a-axis, the length of the HNT on the a-axis L_a is the tube length of the HNT, and the length L_b in the b-axis direction is the perimeter of the crimped layer.

Replace the radius of all layers with the radius of the interlayer of the nanotube, then:

$L_a = 1.2\ \mu m = 1200\ nm$,

$L_b = 2\pi R_{middle} \times b = 2\pi \times (40+10)\ nm \div 2 \times 43 = 6{,}754.4242\ nm$.

As there is no accurate crystal parameters of halloysite, the crystal parameters of halloysite is represented by the crystal parameters of kaolinite . The length of a single crystal is 8.942 Å on the b-axis and 5.154 Å on the a-axis. If a halloysite crystal contains X cells, then

$X = L_a \times L_b \div (8.942\ Å \times 5.154\ Å) = 1200\ nm \times 6{,}754.4242\ nm \div 0.8942\ nm \div 0.5154\ nm \approx 17586949$.

The relative molecular weight M_l of each $Al_4(Si_4O_{10})OH_8$ cell is 516.3204 g/mol. So the relative molecular weight M_2 of an HNT is

$M_2 = 516.3204\ g/mol\ X = 516.3204\ g/mol \times 17586949 = 9{,}080{,}500{,}542.4596\ g/mol$

N_A is the Avogadro constant and $N_A = 6.02 \times 10^{23}$, so the weight m_l of an HNT is

$m_l = M_2 \div N_A = 1.5084 \times 10^{-14}$ g.

(2) The estimate of electrostatic repulsion

According to the, the CEC of HNTs is 40 mmol/100 g, so the amount of substance n_l of charge on an HNT is

$n_l = CEC \times m_l = 40\ mmol/100\ g \times 1.5084 \times 10^{-14}\ g = 6.0336 \times 10^{-18}$ mol.

If Q1 is the charge of an HNT, then

$Q1 = n1\ N_A\ e$, where $e=1.6022 \times 10^{-19}$ C.

The volume V_l of the cylindrical model of MO solution is

$V_l = \pi R_{inner}^2\ L_a = \pi \times (10\ nm)^2 \times 1200\ nm = 3.7699 \times 10^{-22}\ m^3$.

When the concentration C_l of MO is 10 mg/L, the mass m_2 of cylindrical MO is

$m_2 = C_l \times V_l = 10\ mg/L \times 3.7699 \times 10^{-22}\ m^3 = 3.7699 \times 10^{-21}$ g.

The amount of substance M_3 in MO is 327.33 g/mol. The amount of substance n_2 of the charge of the MO solution with a cylindrical model is

$n_2 = m_2 \div M_3 = 3.7699 \times 10^{-21}\ g \div 327.33\ g/mol = 1.1517 \times 10^{-23}$ mol.

When the concentration C_2 of MO is 400 mg/L, the mass m_3 of cylindrical MO is

$m_3 = C_2 \times V_l = 400\ mg/L \times 3.7699 \times 10^{-22}\ m^3 = 1.5080 \times 10^{-19}$ g.

The amount of substance n_2 of the charge of the MO solution with a cylindrical model is

$n_3 = m_3 \div M_3 = 1.5080 \times 10^{-19}$ g $\div$ 327.33 g/mol = 4.0500×10^{-22} mol.

Therefore, the charge quantities Q_2 and Q_3 of MO solutions with concentrations of C_1 and C_2 are:

$Q_2 = n_2 \cdot N_A \cdot e$, $Q_3 = n_3 \cdot N_A \cdot e$.

$$F_K = k\frac{Q_1 Q_2}{r^2} \quad (1)$$

where k is electrostatic force constant (k=8.987551 × 10^9 $N \cdot m^2 \cdot C^{-2}$), r is the distance between point charges ($r=L_a/2$=600 nm).

If the electrostatic forces of 10 mg/L, 400 mg/L MO and an HNT are F_{K1} and F_{K2}, respectively:

$F_{K1} = k\,(Q1 \times Q2) \div r^2 = k\,(n1 \cdot N_A \cdot e \times n_2 \cdot N_A \cdot e) \div L_a/2 = 1.6139 \times 10^{-8}$ N,

$F_{K2} = k\,(Q1 \times Q3) \div r^2 = k\,(n1 \cdot N_A \cdot e \times n_3 \cdot N_A \cdot e) \div L_a/2 = 5.6754 \times 10^{-7}$ N.

2.Calculation of additional pressure

$$\Delta p = \frac{2\sigma}{r} \quad (2)$$

$$r = \frac{R}{\cos\theta} \quad (3)$$

Equation (1) is Laplace equation, where p, σ and r are the additional pressure, surface tension coefficient and radius of curvature of the curved liquid surface, R is the capillary radius and θ is the contact angle. According to Equation 2 and 3, Equation 4 can be obtained:

$$\Delta p = 2\sigma \cdot \frac{\cos\theta}{R} \quad (4)$$

The Laplace equation is the relationship between surface tension, additional pressure, and curvature radius of the curved liquid surface. According to Equation 4, if the additional pressure of capillary action of MO solution in a HNT is to be obtained, the surface tension coefficient of MO solution and the contact angle of MO on the inner surface of HNTs need to be obtained.

Therefore, the contact angles θ_1 and θ_2 of 10 mg/L and 400 mg/L MO solutions on the surface of Al-OH were measured by the method of dosage angle, respectively: $\theta_1 = 15$ °, and $\theta_2 = 19.5$ ° (as shown in Figure S2).

$$\sigma = \frac{\rho g h r}{2\cos\beta} \quad (5)$$

Equation 5 the formula for calculating the surface tension coefficient by the capillary method. Here ρ is the solution density, g is the gravitational acceleration, h is the height at which the capillary also rises, r is the capillary radius and β is the contact angle of the solution on the inner wall of the capillary. And g =9.8 $m \cdot s^{-2}$ and r = 0.25 mm are known. The densities ρ_1 and ρ_2 of 10 and 400 mg/L MO solutions are 1029.64 and 1038.96 kg/m^3, respectively. The height h_1 and h_2 of 10 and 400 mg/L MO solution in capillary are 28 and 26.5 nm, respectively.

According to Equation 5, the closer β is to 0cos β, the larger the calculated σ is. When β = 0, cos β = 1, Equation 6 is obtained.

$$\sigma = \frac{\rho g h r}{2} \quad (6)$$

So the σ_1 and σ_2 of 10 and 400 mg/L MO solution are:

$\sigma_1 = \rho_1 \cdot g \cdot h_1 \cdot r/2 = 3.5317 \times 10^{-2}$ N/m,

$\sigma_2 = \rho_2 \cdot g \cdot h_2 \cdot r/2 = 3.3727 \times 10^{-2}$ N/m.

Therefore, the Δp_1 and Δp_2 of 10 and 400 mg/L MO solution in HNTs are:

$\Delta p_1 = 2\sigma_1 \cdot \cos\theta_1/R = 6.8227 \times 10^6$ N,

$\Delta p_2 = 2\sigma_2 \cdot \cos\theta_2/R = 6.3585 \times 10^6$ N.
